# Human‐Mediated Dispersal Drives Global Invasion of *Halyomorpha halys*: A COI Haplotype and Mantel Test Analysis

**DOI:** 10.1002/ece3.74272

**Published:** 2026-09-01

**Authors:** Xiaoyu Yan, Yu Chen, Dianyu Liu, Zhihan Su, Wenyan Xu, Yichen Wang, Chenxi Liu

**Affiliations:** ^1^ Sino‐American Biological Control Laboratory, Institute of Plant Protection Chinese Academy of Agricultural Sciences Beijing China

**Keywords:** COI, *Halyomorpha halys*, haplotype diversity, human‐mediated dispersal, invasion mechanism, mantel test

## Abstract

The brown marmorated stink bug, *Halyomorpha halys* (Hemiptera: Pentatomidae), is a globally invasive agricultural pest that has rapidly expanded across North America, Europe, and Asia over the past three decades. Understanding the drivers of its invasion success is critical for predicting future spread and informing management. In this study, we assembled a comprehensive mitochondrial COI dataset comprising 1039 sequences from 15 countries across the species' native and introduced ranges, including 119 newly obtained sequences. We identified 65 haplotypes, including eight novel variants, and performed genetic diversity analysis, haplotype network reconstruction, and Mantel tests to quantitatively assess whether patterns of genetic differentiation are consistent with isolation by distance (IBD) or isolation by environment (IBE). Native populations, particularly China and Japan, exhibited high haplotype diversity and pronounced genetic differentiation, suggesting long‐term independent evolution. Introduced populations generally showed reduced diversity consistent with founder effects, with two notable exceptions: Greece and Belgium displayed high haplotype diversity and positive Fu's Fs values, identifying them as potential genetic admixture zones resulting from multiple introductions. The haplotype network revealed two dominant lineages, H1 and H3, with distinct geographic distributions. Mantel tests detected no significant correlations between genetic distance and either geographic distance or climatic dissimilarity (*p* > 0.05). While the limited resolution of a single mitochondrial marker precludes definitive conclusions about the absence of IBD/IBE, these findings are consistent with the hypothesis that frequent and large‐scale human‐mediated dispersal may have overwhelmed signals of natural population structure. By quantitatively decoupling genetic structure from biogeographic templates, this study advances our understanding of invasion mechanisms and underscores the need for biosecurity strategies that target human‐mediated dispersal pathways.

## Graphical abstract

We assembled a global mitochondrial COI dataset of 1039 sequences from 15 countries to assess genetic diversity and test whether patterns of genetic differentiation in the invasive brown marmorated stink bug ( *Halyomorpha halys* ) are consistent with isolation by distance (IBD) or isolation by environment (IBE). While native populations exhibited high haplotype diversity and Greece/Belgium were identified as genetic admixture zones, Mantel tests detected no significant correlations between genetic distance and either geographic or climatic distance. These findings provide a global genetic baseline and suggest that frequent human‐mediated dispersal may have overwhelmed signals of natural population structure.

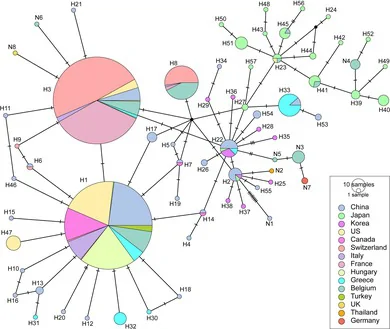

## 1 Introduction

Biological invasions represent a major component of global environmental change, with significant ecological and economic consequences (Simberloff et al. 2013). A fundamental question in invasion biology is whether the spread of non‐native species is primarily governed by natural dispersal barriers—such as geographic distance (isolation by distance, IBD) and climatic suitability (isolation by environment, IBE)—or whether human‐mediated transportation has become sufficiently pervasive to override these natural constraints (Wang and Bradburd 2014). Resolving this question is essential for predicting invasion risks and developing evidence‐based management strategies.

The brown marmorated stink bug, (Hemiptera: Pentatomidae), provides an excellent model to address this question. Native to East Asia (China, Japan, and the Korean Peninsula), this polyphagous pest feeds on over 300 host plants, causing severe economic losses to fruits, vegetables, and ornamentals (Lee et al. 2013; Yan et al. 2021). Since its first detection in Pennsylvania, USA in 1996 (Hoebeke and Carter 2003), *Halyomorpha halys* has rapidly expanded its range across North America, Europe, and more recently into South America (Chile), establishing self‐sustaining populations in dozens of countries (Valentin et al. 2017; Leskey and Nielsen 2018). Its global invasion success has been attributed to a combination of factors, including climatic suitability (Zhu et al. 2012; Kriticos et al. 2017), its propensity to hitchhike on traded goods (Vandervoet et al. 2019), and the potential for secondary spread from already‐invaded regions—the so‐called bridgehead effect (Lombaert et al. 2010; Valentin et al. 2017).

The mitochondrial cytochrome c oxidase subunit I (COI) gene, characterized by maternal inheritance, moderate evolutionary rate, and ease of amplification, has been widely used in insect population genetics and invasion pathway tracking (Hebert et al. 2003). Previous genetic studies have made significant strides in tracing the invasion routes of *H. halys* . Using mitochondrial DNA (mtDNA) sequences, Valentin et al. (2017) employed Approximate Bayesian Computation (ABC) to reconstruct the global invasion network, revealing multiple independent introductions from China into North America and Europe, as well as a likely bridgehead event from the eastern USA to Italy. Subsequent work by Yan et al. (2021) expanded the dataset and identified numerous novel COI and COII haplotypes, confirming the complex, multi‐source nature of the invasion. More recently, population genomic analyses using double‐digest RADseq (Parvizi et al. 2023) uncovered high levels of admixture among invasive populations and comparable genomic diversity between native and invasive ranges, suggesting that multiple introductions have mitigated founder effects. Remarkably, Parvizi et al. (2023) also identified outlier SNPs located near genes associated with insecticide resistance and olfaction in invasive populations, raising the possibility that standing genetic variation may contribute to local adaptation. Building on this genomic resource, Boscolo Agostini et al. (2025) applied Approximate Bayesian Computation (ABC) to the same ddRAD dataset and explicitly tested alternative invasion scenarios, providing quantitative support for multiple waves of migration from East China to invaded territories and identifying bridgehead effects from North America to the Near East and from Europe to South America (Boscolo Agostini et al. 2025). Complementing these findings, Ozdemir et al. (2025) demonstrated that nuclear markers (Start Codon Targeted polymorphisms) provide superior resolution for detecting fine‐scale genetic structure in recently established populations, highlighting the limitations of relying solely on mtDNA.

Despite these advances, a critical gap remains: no study has yet quantitatively tested, at a global scale, whether the observed genetic differentiation among global *H. halys* populations is primarily driven by IBD or IBE. Such tests are essential for distinguishing between natural dispersal mechanisms and human‐mediated transport as the dominant drivers of invasion. If IBD or IBE were significant, we would expect populations from geographically close or climatically similar regions to be genetically more similar, supporting natural expansion models. Conversely, if human activities randomly shuffle individuals across the globe, we would predict no significant correlations between genetic distance and either geographic or climatic distance.

The Mantel test, a classical method for assessing matrix correlations, provides a robust framework for addressing this question (Xue et al. 2025). By integrating comprehensive genetic data with geographic and climatic distance matrices, we can quantitatively evaluate the relative importance of these factors in shaping global population structure. Here, we assemble the largest mtDNA COI dataset for *H. halys* to date, comprising 1039 sequences from 15 countries across its native and introduced ranges. We combine genetic diversity analysis, haplotype network reconstruction, and Mantel tests to achieve two primary objectives: (i) to provide a comprehensive inventory of global COI haplotype diversity, including the identification of novel haplotypes and potential admixture zones; and (ii) to conduct the first global‐scale, quantitative test of whether patterns of genetic differentiation are consistent with IBD and IBE models. By doing so, we aim to establish a genetic baseline for future genomic studies and contribute to a more nuanced understanding of the drivers shaping *H. halys* invasion success.

While genome‐wide approaches provide superior resolution for demographic reconstruction and local adaptation (Parvizi et al. 2023; Boscolo Agostini et al. 2025), mitochondrial COI remains well‐suited for addressing questions of broad‐scale phylogeography and testing macroecological patterns such as IBD and IBE, due to its maternal inheritance, moderate evolutionary rate, and extensive public availability of geo‐referenced sequences across the species' global range. We acknowledge that the use of a single mitochondrial marker and uneven geographic sampling across some regions represent inherent limitations of this study. However, given the extensive geographic coverage and large sample size, this dataset provides a valuable foundation for generating hypotheses about invasion drivers that can be tested in future studies using higher‐resolution genomic markers.

## 2 Materials and Methods

### 2.1 Data Collection and Haplotype Identification

The COI sequence data of *H. halys* analyzed in this study were derived from two sources. The first part integrated the global population genetic dataset of *H. halys* published by Valentin et al. (2017), comprising 920 samples from established populations across North America, Europe, and Asia. This dataset contained 658 bp sequences of the COI barcoding region, with sampling spanning from 2004 to 2016 (Table S1). The second part consisted of 119 newly downloaded sequences from NCBI GenBank and the Barcode of Life Data System (BOLD) published after 2021. These sequences had clear geographic origin information and were ≥ 600 bp in length, primarily originating from Belgium, Turkey, South Korea, and other countries (Table S2). The 920 sequences (658 bp) from Valentin et al. (2017) were combined with the 119 newly added sequences and aligned using MEGA v11, then trimmed to a uniform length of 657 bp by removing one redundant site introduced during alignment.

**FIGURE 1:**
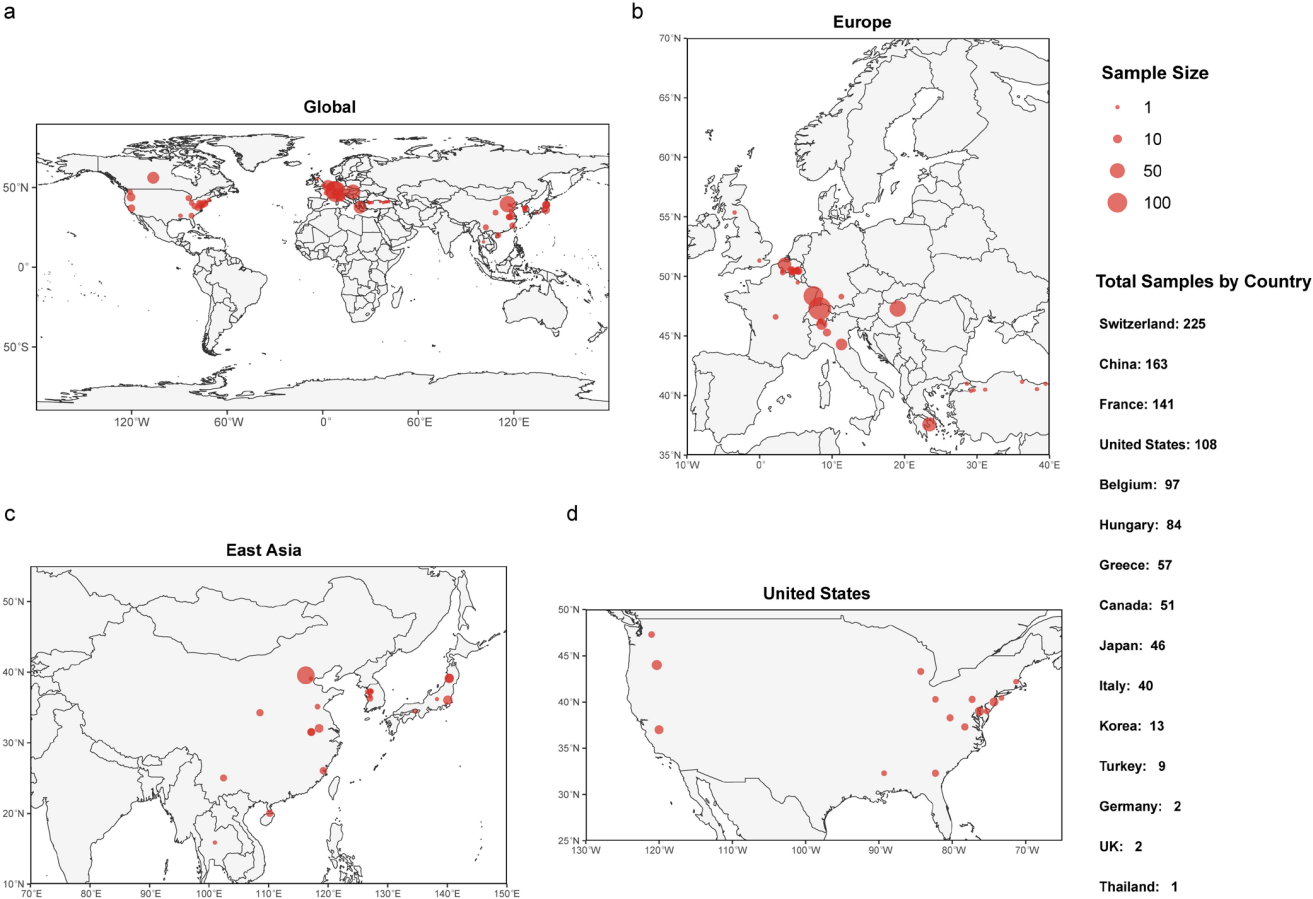
Geographic distribution of *Halyomorpha halys* mtDNA COI haplotypes worldwide. Dot sizes represent sample sizes. (a) Global distribution of all sampling sites. (b) Distribution of samples in Europe. (c) Distribution of samples in East Asia. (d) Distribution of samples in the United States.

Following the published COI haplotype nomenclature system for *H. halys* (Valentin et al. 2017; Yan et al. 2021), haplotypes were assigned as follows: sequences identical to previously reported haplotypes retained their original haplotype names; sequences that did not match any in the published nomenclature system were considered novel haplotypes and were sequentially named N1 to N8 with the prefix “N”. The final dataset comprised 1039 COI sequences from 15 countries, including: China (163), Japan (46), South Korea (13), USA (108), Canada (51), Switzerland (225), Italy (40), France (141), Hungary (84), Greece (57), Belgium (97), Turkey (9), United Kingdom (2), Thailand (1), and Germany (2) (Figure 1).

### 2.2 Genetic Diversity Analysis

Population genetic diversity was assessed using DnaSP v6 (Rozas et al. 2017) based on the number of haplotypes (Hn), haplotype diversity (Hd), and nucleotide diversity (π) to quantify the degree of genetic diversity. To detect deviations from neutral evolution and infer population demographic history, Fu's Fs neutrality test (Fu 1997) was performed for each population. The test statistics were calculated using the hfufs package in R, which employs an asymptotic estimation algorithm suitable for large sample sizes. Fu's Fs is based on the probability of observing a number of haplotypes in a random sample less than or equal to the observed value (k), given the observed average number of nucleotide differences (θ). Statistical significance was evaluated using 1000 coalescent simulations. Populations with sample sizes less than 4 were excluded from analysis due to insufficient statistical power (marked as NA). For populations with a single haplotype (k = 1), Fu's Fs was set to 0 and the *p*‐value to 1.0, indicating neutral evolution. When Fu's Fs was negative with *p* < 0.05, the population was inferred to have experienced significant expansion.

Genetic differentiation among *H. halys* populations was estimated using the fixation index (*F*_ST_). Based on the pairwise *F*_ST_ distance matrix, principal coordinate analysis (PCoA) was employed to visualize genetic relationships among populations. PCoA was performed in R using the pcoa function from the ape package (Paradis and Schliep 2019). A phylogenetic network of *H. halys* COI haplotypes was constructed using the TCS algorithm implemented in PopART software (Clement et al. 2002; Leigh and Bryant 2015).

### 2.3 Distance Matrix Construction and Mantel Test

To assess the relationships between genetic differentiation of *H. halys* and both environmental factors and geographic distance, three distance matrices were constructed and subjected to Mantel tests. The genetic distance matrix was calculated based on COI sequences of *H. halys* populations from 15 countries using Nei's standard genetic distance (Nei 1972) implemented in the PopGenReport package in R, estimating the degree of genetic differentiation among populations. The geographic distance matrix was constructed by integrating sampling site coordinates and sample size weights. The raw data contained sampling site information from 15 countries (Table S3). The weighted geographic center for each country was calculated using the weighted average method. Based on the coordinates of each country's weighted geographic center, the spherical distance between pairs of countries was calculated using the Haversine formula to account for the effect of Earth's curvature on distance measurement. The climatic distance matrix was derived from bioclimatic variables extracted from the WorldClim database version 2.1 (http://www.worldclim.org) (Fick and Hijmans 2017) for each sampling site. To select environmental predictors for the IBE analysis, we adopted the six‐variable set established by Marchioro and Krechemer (2024) and Xue et al. (2025), who screened 20 bioclimatic variables plus elevation for multicollinearity using global‐scale occurrence data and retained variables with pairwise |*r*| < 0.8. Given that our sampling locations span climatically diverse regions across Asia, Europe, and North America—comparable to the global gradient used in those studies—this variable set is appropriate for our IBE analysis. The six variables are: annual mean temperature (Bio1), temperature annual range (Bio7), annual precipitation (Bio12), precipitation of the driest month (Bio14), precipitation seasonality (Bio15), and elevation (Marchioro and Krechemer 2024; Xue et al. 2025). Climatic variable values for 75 sampling sites were extracted using the terra package in R, with missing values handled through a multi‐level buffer interpolation strategy (Table S4). Using sample sizes at each sampling site as weights, the weighted arithmetic means of the six climatic variables were calculated for each country. After *Z*‐score standardization, the Euclidean distance between pairs of countries was calculated as the climatic dissimilarity matrix. Mantel tests were performed using the vegan package in R with 9999 permutations to evaluate Pearson and Spearman correlations between the genetic distance matrix and both the geographic distance and climatic distance matrices.

In addition to the global Mantel test across all 15 countries, we performed partitioned Mantel tests for three geographic regions: (i) East Asia (China, Japan, South Korea; native range), (ii) Europe (Switzerland, Italy, France, Hungary, Greece, Belgium, United Kingdom, Germany; invaded range), and (iii) the United States (15 state‐level populations; invaded range). For each region, we tested both IBD and IBE. Partial Mantel tests (genetic distance ~ climatic distance | geographic distance) were also conducted to isolate the independent effect of climate after accounting for geography. All partitioned tests followed the same procedure as the global analysis with 9999 permutations in the vegan R package.

## 3 Results

### 3.1 Sequence Characteristics and Global Haplotype Distribution

A total of 1039 COI sequences of *H. halys* from 15 countries were analyzed, trimmed to a uniform length of 657 bp with no indels or stop codons, confirming functional mitochondrial origin. We identified 65 distinct haplotypes, comprising 57 previously reported haplotypes (prefix H) and eight novel haplotypes (N1–N8). Novel haplotypes exhibited strong geographic specificity: N1 was unique to China, N2 to Thailand, N3–N6 to Belgium, N7 to Germany, and N8 to the United Kingdom (Table 1). At the country level, China harbored the highest haplotype diversity with 29 haplotypes, followed by Japan (20) and South Korea (9) (Table 1). Invasive populations generally exhibited low haplotype numbers, consistent with founder effects: USA (5), Canada (3), Switzerland (4), Italy (3), France (3), Hungary (2), Turkey (1), United Kingdom (2), and Germany (1). However, Greece and Belgium deviated markedly from this pattern, displaying 7 and 9 haplotypes respectively. Notably, Belgium harbored four novel haplotypes (N3–N6), suggesting ongoing local differentiation or sustained gene flow from multiple sources.

**TABLE 1:** Sample sizes and haplotype distribution of *Halyomorpha halys* in 15 countries.

Country	Samples	Haplotype
China	163	H1(92), H2(7), H3(19), H4(1), H5(1), H6(1), H7(1), H10(1), H11(1), H12(1), H13(3), H14(1), H15(1), H16(1), H17(5), H18(1), H19(1), H20(1), H21(1), H22(8), H26(2), H33(3), H34(1), H45(1), H46(1), H53(2), H54(3), H55(1), N1(1)
Japan	46	H1(1), H2(1), H22(1), H23(2), H24(1), H27(2), H39(4), H40(6), H41(5), H42(1), H43(1), H44(2), H45(5), H48(1), H49(1), H50(1), H51(8), H52(1), H56(1), H57(1)
Korea	13	H2(1), H22(5), H25(1), H28(1), H29(1), H35(1), H36(1), H37(1), H38(1)
US	108	H1(82), H3(13), H7(1), H23(1), H47(11)
Canada	51	H1(49), H6(1), H14(1)
Switzerland	225	H1(2), H3(191), H8(31), H9(1)
Italy	40	H1(32), H3(7), H8(1)
France	141	H1(1), H3(138), H8(2)
Hungary	84	H1(83), H3(1)
Greece	57	H1(18), H3(4), H22(2), H30(1), H31(1), H32(8), H33(23)
Belgium	97	H1(33), H3(21), H8(25), H23(1), H41(2), N3(8), N4(5), N5(1), N6(1)
Turkey	9	H1(9)
UK	2	H3(1), N8(1)
Thailand	1	N2(1)
Germany	2	N7(2)

*Note:* The number in parentheses indicates the count of a haplotype in that country.

### 3.2 Genetic Diversity Analysis Based on COI

Genetic diversity indices revealed stark contrasts between native and invasive populations (Table 2). Native populations exhibited high diversity: Japan showed the highest haplotype diversity (Hd = 0.9285, π = 0.00425), followed by South Korea (Hd = 0.8718, π = 0.00281) and China (Hd = 0.6646, π = 0.00233). Outside the native range, the highest diversity was observed in Belgium (Hd = 0.7687, π = 0.00394) and Greece (Hd = 0.7237, π = 0.00356), comparable to or exceeding levels in some native populations. In contrast, most invasive populations showed severely reduced diversity, with Hd values below 0.4 in the USA, Switzerland, Italy, and France, and near‐zero in Canada (0.0776), Hungary (0.0239), and Turkey (0).

**TABLE 2:** Genetic diversity analysis of *Halyomorpha halys* based on COI sequences.

Country	Samples	Hn	Hd	π	Fu's Fs	*p*
China	163	29	0.6646	0.00233	−26.1816	0.071
Japan	46	20	0.9285	0.00425	−11.4769	0.062
Korea	13	9	0.8718	0.00281	−5.6554	0.073
US	108	5	0.4022	0.00074	−1.488	0.123
Canada	51	3	0.0776	0.000119	−3.0645	0.174
Switzerland	225	4	0.2615	0.000767	0.0603	0.163
Italy	40	3	0.3372	0.000652	−0.0724	0.174
France	141	3	0.0421	0.000107	−2.8463	0.174
Hungary	84	2	0.0239	0.000036	−2.075	0.256
Greece	57	7	0.7237	0.003563	1.0843	0.132
Belgium	97	9	0.7687	0.003935	0.851	0.118
Turkey	9	1	0	0	0	1
UK	2	2	1	0.003044	NA	NA
Thailand	1	1	0	0	NA	NA
Germany	2	1	0	0	NA	NA

*Note:* The total sample size (*N*), number of haplotypes (Hn), haplotype diversity (Hd), nucleotide diversity (π), and Fu's Fs values are listed for each country.

Neutrality tests provided insights into demographic history. Chinese and Japanese populations showed large negative Fu's Fs values (China: −26.18; Japan: −11.48), indicating historical population expansion in the native range. Among invasive populations, the USA (−1.49), Canada (−3.06), France (−2.85), and Hungary (−2.08) exhibited negative values, suggesting post‐introduction expansion. Critically, Greece (1.08) and Belgium (0.85) displayed positive Fu's Fs values, a signal consistent with population bottlenecks or recent admixture between genetically distinct source populations. This demographic signal, combined with their elevated haplotype diversity, provides statistical support for classifying Greece and Belgium as genetic admixture zones.

**FIGURE 2:**
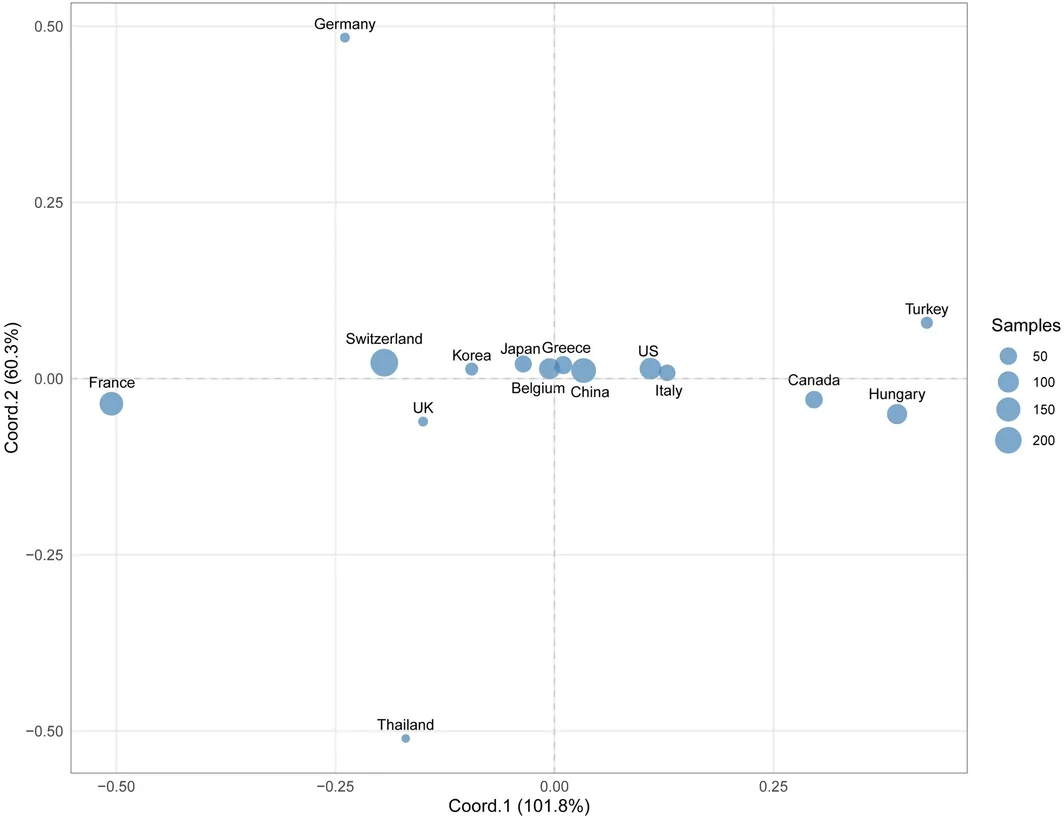
Principal coordinate analysis (PCoA) based on pairwise genetic distances of *Halyomorpha halys* populations. Dot sizes represent sample sizes. The *X*‐axis shows the values of Coord.1, and the *Y*‐axis shows the values of Coord.2. The percentages of variation explained by Coord.1 and Coord.2 are 101.8% and 60.3%, respectively.

Principal Coordinate Analysis (PCoA) based on *F*_ST_ values revealed that the Italian *H. halys* population was closely related to the US population. The 15 country populations were divided into two distinct genetic clusters. The positive Coord.1 group included China (0.033), USA (0.110), Canada (0.296), Italy (0.129), Hungary (0.391), Greece (0.010), and Turkey (0.425), with H1 as the dominant haplotype in these countries. The negative Coord.1 group included Japan (−0.036), South Korea (−0.094), Switzerland (−0.194), France (−0.506), Belgium (−0.006), United Kingdom (−0.150), Thailand (−0.170), and Germany (−0.239), with H3, H8, and other haplotypes predominating. Notably, Turkey showed close genetic distance to Hungary and Canada, suggesting the Turkish population may be closely related to populations of Chinese or North American origin. France was relatively close to Western European populations such as Switzerland and Belgium, consistent with its geographic location and haplotype composition (Figure 2). The PCoA results were highly consistent with the haplotype distribution pattern, further supporting the inference of at least two main genetic lineages in global *H. halys* populations: one lineage represented by H1 widely distributed in China, North America, and parts of Southern Europe; another lineage represented by H3, H8, and other haplotypes mainly distributed in Japan, South Korea, and Western European countries.

**FIGURE 3:**
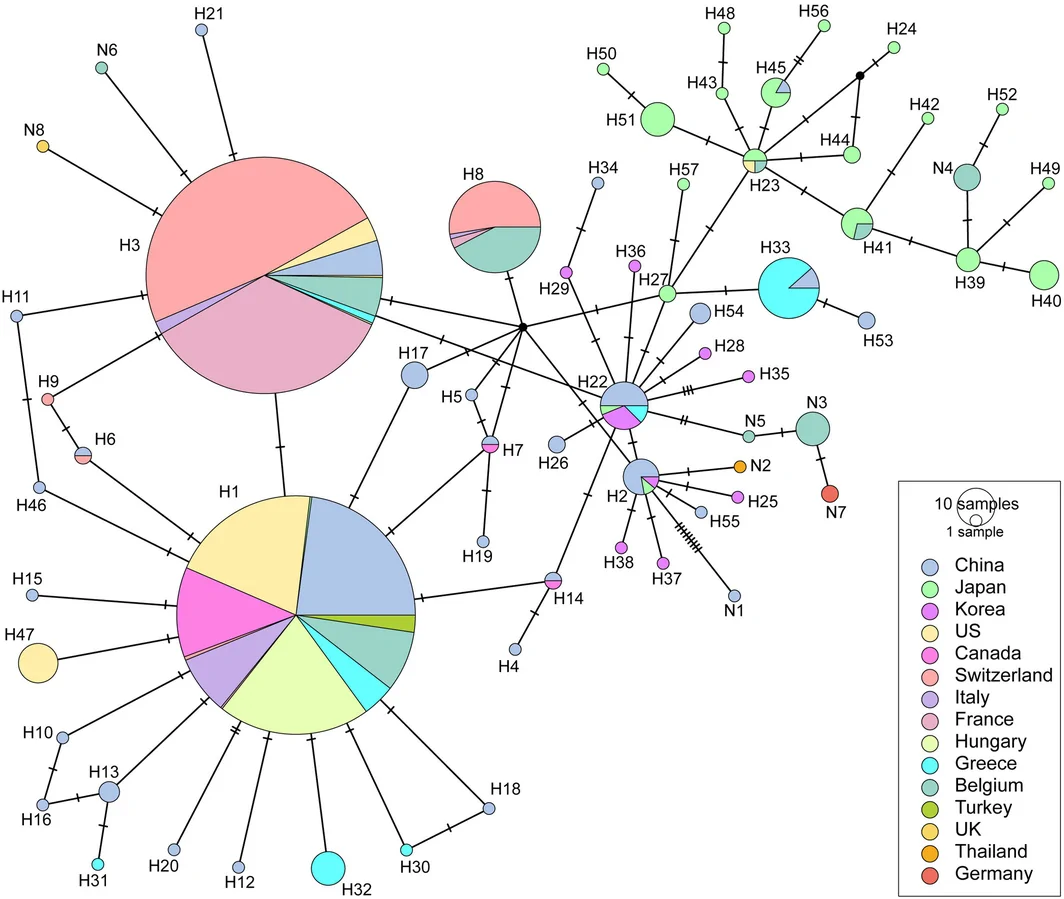
COI haplotype network of *Halyomorpha halys* . Each circle represents a haplotype. The size of each circle is proportional to the haplotype frequency. Colors represent the countries where samples were collected. Hash marks on each line represent one base pair difference.

Building upon the haplotype network of Valentin et al. (2017), haplotypes identified from newly added sequences published after 2021 were incorporated (Figure 3). Consistent with previous reports, global *H. halys* populations exhibited two main genetic lineages, H1 and H3, corresponding to two dispersal routes: Asia‐North America‐Southern Europe and Asia‐Western Europe‐Central Europe. Although China and Japan are geographically adjacent native regions, they shared almost no haplotypes, showing significant genetic differentiation, suggesting long‐term isolation and independent evolution of the two populations. Critically, the network revealed several populations with complex haplotype compositions that deviate from the simple core‐lineage pattern. Greece harbored seven haplotypes representing a mixture of multiple lineages, including H1, H3, H22, H30–H33, and H41. Belgium displayed even greater complexity, with nine haplotypes encompassing H1, H3, H8, H23, H41, and four novel haplotypes (N3–N6). The network positions of these haplotypes—connecting to both H1 and H3 lineages—provide visual evidence for secondary contact between previously isolated genetic lineages. These regions may have experienced multiple independent invasion events or now function as secondary contact zones where populations from distinct sources converge and potentially hybridize.

### 3.3 Mantel Tests Reject Isolation by Distance and Environment

**FIGURE 4:**
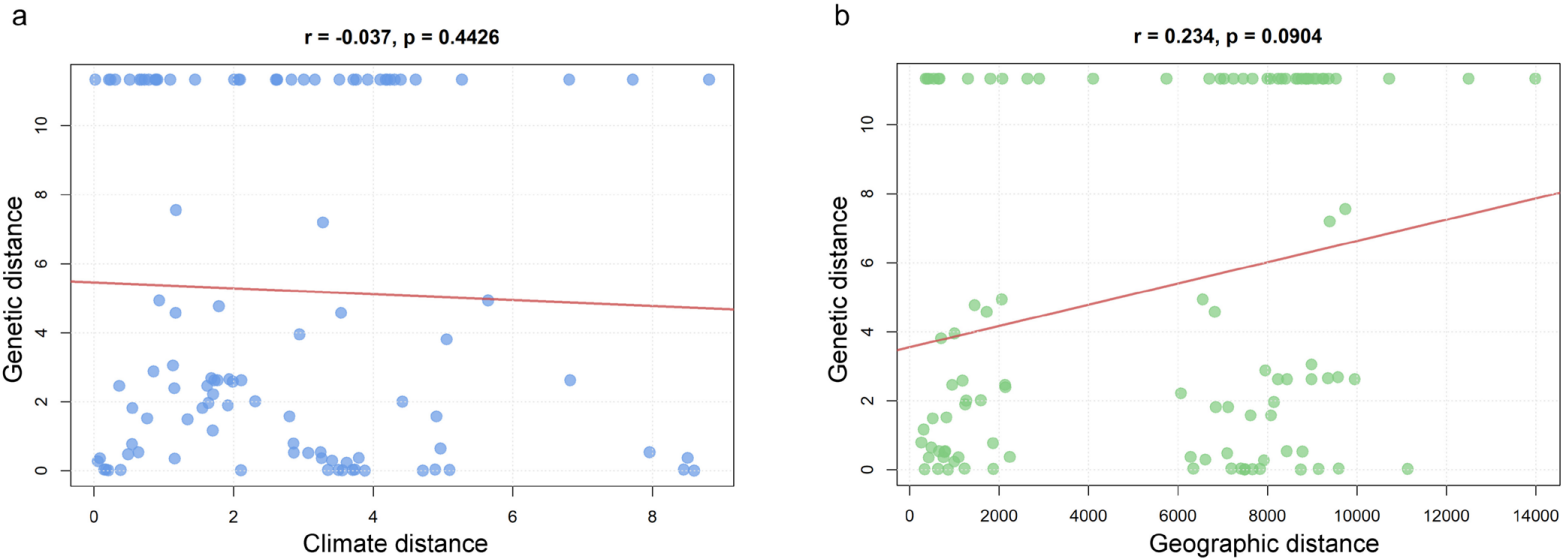
Mantel test scatter plots showing the relationships between genetic distance and both climatic distance and geographic distance. (a) Genetic distance vs. climatic distance; (b) Genetic distance vs. geographic distance.

To quantitatively assess the drivers of genetic differentiation, we performed Mantel tests (9999 permutations) correlating Nei's genetic distance with geographic distance and climatic distance matrices (Figure 4). The IBE test revealed no significant correlation between genetic distance and climatic dissimilarity (Pearson's *r* = −0.0366, *p* = 0.4426; Spearman's *ρ* = −0.0581, *p* = 0.5753). The IBD test also showed no significant correlation between genetic distance and geographic distance (Pearson's *r* = 0.2336, *p* = 0.0904; Spearman's *ρ* = 0.2262, *p* = 0.0957). Although the IBD correlation coefficients were positive and approached the conventional significance threshold, they did not reach statistical significance.

Collectively, these Mantel test results demonstrate that the global population genetic structure of *H. halys* is not significantly correlated with either geographic distance or environmental dissimilarity. These findings indicate that neither IBD nor IBE alone can explain the current patterns of genetic differentiation. The failure of both classical models to gain support points to the involvement of factors beyond natural dispersal mechanisms—specifically, human‐mediated long‐distance dispersal—as the dominant force shaping the global invasion of *H. halys* , effectively overcoming the constraints of geographic and environmental distances on gene flow.

### 3.4 Partitioned Mantel Tests

**FIGURE 5:**
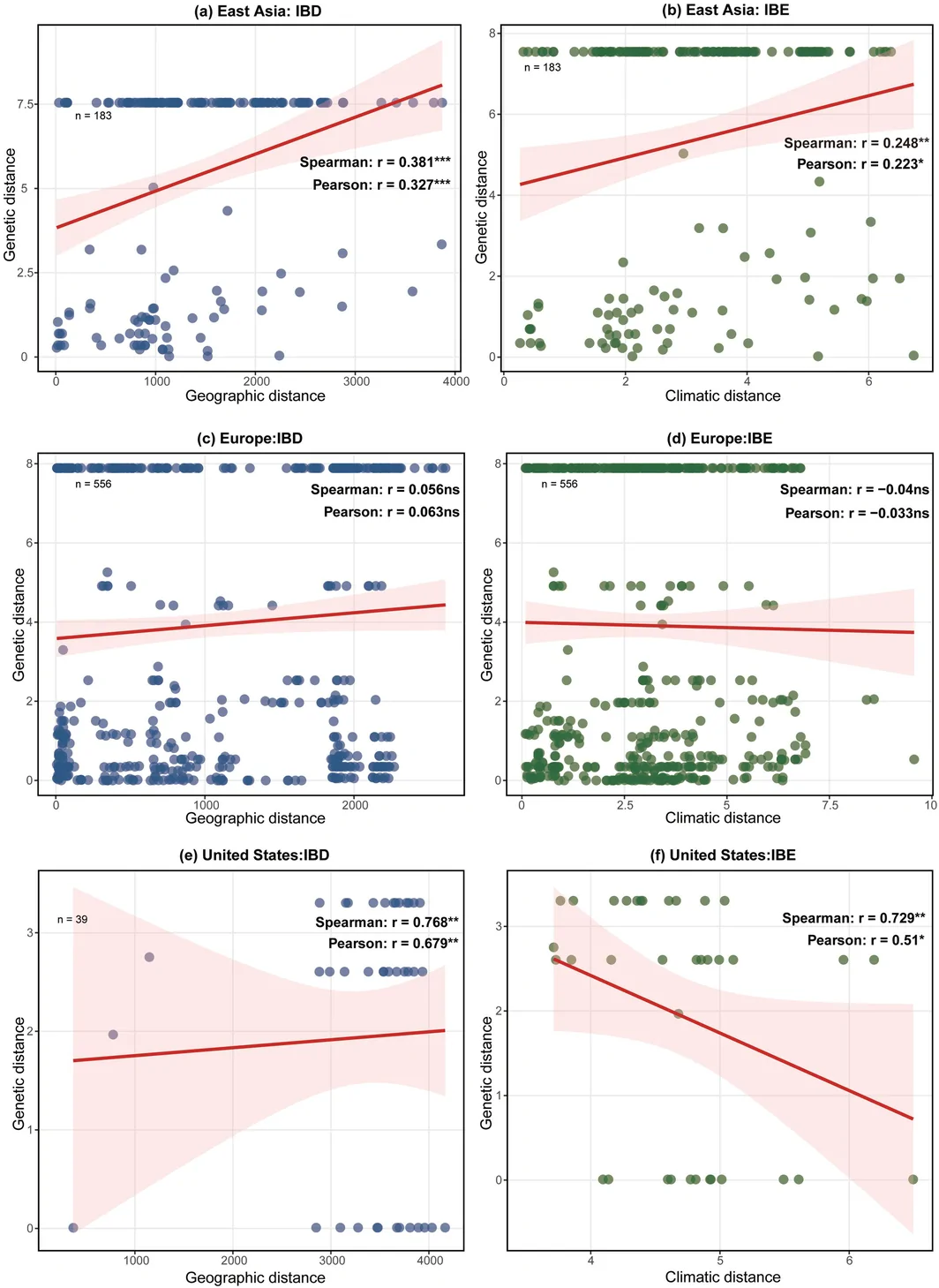
Partitioned Mantel test scatter plots for East Asia (native range), Europe (invaded), and the United States (invaded, state‐level populations). (a) East Asia: Genetic distance vs. climatic distance; (b) East Asia: Genetic distance vs. geographic distance; (c) Europe: Genetic distance vs. climatic distance; (d) Europe: Genetic distance vs. geographic distance; (e) United States: Genetic distance vs. climatic distance; (f) United States: Genetic distance vs. geographic distance. Spearman's *ρ* and associated *p*‐values are shown in each panel. Significance levels: *p < 0.05, **p < 0.01, ***p < 0.001; ns, not significant. Shaded areas represent 95% confidence intervals.

To explore whether IBD or IBE signals differ between native and invaded ranges, we performed partitioned Mantel tests for East Asia, Europe, and the United States (state‐level populations). In the native East Asian range (China, Japan, South Korea), both IBD and IBE were significant: IBD (Spearman's *r* = 0.381, *p* < 0.001; Pearson's *r* = 0.327, *p* < 0.001) and IBE (Spearman's *r* = 0.248, *p* = 0.009; Pearson's *r* = 0.223, *p* = 0.012). The partial Mantel test (genetic distance ~ climatic distance | geographic distance), however, was not significant (Spearman's *r* = −0.065, *p* = 0.753; Pearson's *r* = −0.040, *p* = 0.665), indicating that the observed IBE signal in East Asia is largely confounded by geographic proximity rather than reflecting an independent effect of climate (Figure 5a,b). In invaded Europe, neither IBD (Spearman's *r* = 0.056, *p* = 0.205; Pearson's *r* = 0.063, *p* = 0.201) nor IBE (Spearman's *r* = −0.040, *p* = 0.645; Pearson's *r* = −0.033, *p* = 0.595) was significant. Similarly, the partial Mantel test was not significant (Spearman's *r* = −0.116, *p* = 0.901; Pearson's *r* = −0.103, *p* = 0.832), indicating that climate does not exert an independent effect on genetic structure in Europe after accounting for geography (Figure 5c,d). For the United States (state‐level populations, *n* = 15 states), both IBD and IBE were significant: IBD (Spearman's *r* = 0.768, *p* = 0.001; Pearson's *r* = 0.679, *p* = 0.003) and IBE (Spearman's *r* = 0.729, *p* = 0.002; Pearson's *r* = 0.510, *p* = 0.024). However, the partial Mantel test was not significant (Spearman's *r* = 0.296, *p* = 0.066; Pearson's *r* = −0.059, *p* = 0.603), indicating that the IBE signal in the United States is confounded by geographic distance rather than reflecting an independent climatic effect (Figure 5e,f). These results suggest that natural dispersal barriers operate in the native range—consistent with natural biogeographic processes—but their global‐scale signal is lost in invaded regions, likely due to human‐mediated long‐distance dispersal. The significant IBD and IBE signals in the United States are largely driven by geographic structure rather than independent climatic effects.

## 4 Discussion

In this study, we assembled the largest mitochondrial COI dataset for *H. halys* to date and employed Mantel tests to quantitatively evaluate the drivers of its global invasion. Our results provide compelling evidence that the current genetic structure of *H. halys* populations worldwide is not significantly correlated with either geographic distance or climatic dissimilarity. This absence of both IBD and IBE signals represents a critical departure from patterns expected under natural dispersal scenarios and offers the strongest quantitative evidence to date that natural dispersal barriers are not the primary architects of *H. halys* 's global population structure. Crucially, these findings are highly consistent with the ABC‐based demographic reconstruction of Boscolo Agostini et al. (2025), who inferred multiple independent waves of migration from East China to invaded territories and estimated that the initial invasion into North America occurred approximately 73 years ago—well before the first documented detection in 1996. Together, these complementary lines of evidence—demographic reconstruction (ABC) and mechanistic testing (Mantel)—converge on a unified conclusion: human‐mediated dispersal has overridden natural biogeographic constraints as the dominant driver of global invasion.

The partitioned Mantel tests further illuminate this pattern. In the native East Asian range, significant IBD (Spearman's *ρ* = 0.381, *p* < 0.001; Pearson's *r* = 0.327, *p* < 0.001) and IBE (Spearman's *ρ* = 0.248, *p* = 0.009; Pearson's *r* = 0.223, *p* = 0.012) signals indicate that natural dispersal barriers still shape genetic structure in the absence of extensive human‐mediated transport. In the United States, significant IBD (Spearman's *ρ* = 0.768, *p* = 0.001; Pearson's *r* = 0.679, *p* = 0.003) and IBE (Spearman's *ρ* = 0.729, *p* = 0.002; Pearson's *r* = 0.510, *p* = 0.024) signals—but a non‐significant partial IBE (Spearman's *ρ* = 0.296, *p* = 0.066; Pearson's *r* = −0.059, *p* = 0.603)—suggest that early invasion history still reflects geographic spread from initial introduction points, with climate playing no independent role once geography is accounted for. In Europe, however, neither IBD (Spearman's *ρ* = 0.056, *p* = 0.205; Pearson's *r* = 0.063, *p* = 0.201) nor IBE (Spearman's *ρ* = −0.040, *p* = 0.645; Pearson's *r* = −0.033, *p* = 0.595) was significant, indicating that multiple, repeated introductions from diverse sources have completely overwritten the signal of natural dispersal. This gradient—from significant natural signals in the native range to their progressive attenuation in the United States and complete absence in Europe—provides compelling evidence that human‐mediated dispersal progressively overrides natural biogeographic constraints as invasion history unfolds.

Consistent with previous studies (Xu et al. 2014; Yan et al. 2021), the Chinese population harbored the highest number of haplotypes, supporting East Asia, particularly China, as the native center of origin for *H. halys* . Although the Japanese population exhibited even higher haplotype diversity, it shared almost no haplotypes with China and formed an independent cluster in the TCS network. This pronounced genetic differentiation between geographically adjacent native regions aligns with observations by Yan et al. (2021) and Parvizi et al. (2023), and likely results from long‐term geographic isolation (oceanic barriers) coupled with stringent quarantine measures that restrict contemporary gene flow.

Invasive populations in North America and Europe generally exhibited low haplotype diversity, retaining only a fraction of the genetic diversity present in native populations—a pattern consistent with founder effects (Dlugosch and Parker 2008). However, the absence of IBD/IBE signals suggests that these founder effects have been repeatedly mitigated by multiple, independent introductions from diverse native sources. This interpretation aligns closely with the Approximate Bayesian Computation (ABC) analyses of Valentin et al. (2017), who reconstructed multiple out‐of‐China introductions into North America and Europe, as well as secondary bridgehead events from the USA to Italy. Our Mantel test results provide the underlying mechanistic explanation for these complex invasion routes: human activities randomly shuffle genetic lineages across continents, thereby eroding any expected correlations with geography or climate.

Notably, Greece and Belgium deviated from the general pattern of low diversity, displaying remarkably high haplotype diversity accompanied by positive Fu's Fs values. These characteristics, coupled with the detection of four novel haplotypes in Belgium (N3–N6) and a mixture of multiple lineages in Greece, identify these regions as genetic admixture zones or secondary contact zones. Such zones are of particular evolutionary significance, as they may facilitate the generation of novel adaptive combinations from standing genetic variation (Lombaert et al. 2010). Population genomic analyses by Parvizi et al. (2023) identified outlier SNPs in invasive populations located near genes associated with insecticide resistance and olfaction, suggesting that adaptive evolution may be acting on standing variation. The admixture zones we have identified in Greece and Belgium could be precisely where such adaptive variation is generated and tested, warranting intensive monitoring and further genomic investigation. The high genetic diversity observed in these regions, combined with their positive Fu's Fs values, suggests that local selective pressures may still be acting on standing genetic variation after arrival, a possibility consistent with the findings of Parvizi et al. (2023). Future studies should investigate whether the admixture zones identified here exhibit signatures of local adaptation to climatic or agricultural practices, potentially using genome‐wide approaches to disentangle the effects of demography and selection.

The non‐significant IBD/IBE results alone do not directly prove human‐mediated dispersal; rather, they refute the alternative hypothesis that natural dispersal barriers explain the observed genetic structure. However, multiple lines of indirect evidence support human‐mediated dispersal as the most parsimonious explanation. First, the transcontinental distribution of dominant haplotypes H1 and H3—found in East Asia, North America, and Europe—is difficult to explain by natural dispersal across oceans. Second, historical interception data have documented *H. halys* on shipped goods, including vehicles, timber, and agricultural products (Hoebeke and Carter 2003; Vandervoet et al. 2019). Third, the admixture zones we identified in Greece and Belgium coincide with major international shipping ports (e.g., Piraeus, Antwerp), consistent with human‐mediated introduction. Fourth, the positive Fu's Fs values in these same regions suggest recent demographic changes consistent with admixture following multiple introductions, rather than natural range expansion. Fifth, ABC analyses of genomic data have independently reconstructed multiple wave invasions and bridgehead effects (Boscolo Agostini et al. 2025; Valentin et al. 2017). The transcontinental distribution of H1 and H3 haplotypes revealed in the TCS network implicates human‐mediated transport—particularly through international trade in agricultural products, timber, and shipping containers—as the primary driver of intercontinental dispersal. This interpretation is corroborated by historical records and interception data (Hoebeke and Carter 2003; Vandervoet et al. 2019), and our quantitative results now provide robust statistical support for the “human‐mediated dispersal” hypothesis.

Several limitations of this study should be acknowledged. First, small sample sizes in certain countries (Turkey, United Kingdom, Thailand, Germany) may have led to underestimation of true genetic diversity. Second, as a single mitochondrial marker, COI reflects only maternal lineage history and cannot fully represent the evolutionary history of the nuclear genome. Indeed, recent genome‐wide studies using ddRAD data have provided higher‐resolution insights into the invasion history of *H. halys* , including quantitative estimates of introduction times (approximately 73 years ago into North America and 32 years ago into Europe) and bridgehead effects from North America to the Near East and from Europe to South America (Boscolo Agostini et al. 2025; Parvizi et al. 2023). However, these genomic studies did not quantitatively test the predictions of IBD and IBE at a global scale—a gap that our study fills. The high‐resolution population structure recently detected in Turkish populations using Start Codon Targeted (SCoT) markers (Ozdemir et al. 2025) highlights this limitation: while mitochondrial DNA is excellent for tracing broad, ancient maternal lineages, it may miss recent, fine‐scale genetic diversification driven by local adaptation or admixture. Future global studies should integrate nuclear markers, such as SCoTs or genome‐wide SNPs, to simultaneously test for IBD/IBE signatures and reconstruct demographic histories. Third, while our study employs Mantel tests to test IBD and IBE, we acknowledge that more sophisticated approaches (e.g., partial Mantel tests, multiple matrix regression, or landscape genetic analyses) could provide additional insights. However, for the purpose of testing the presence or absence of global‐scale IBD/IBE signals, the Mantel test remains a widely accepted and appropriate first‐line method. Fourth, environmental distances were derived from macroclimatic data obtained from WorldClim, which does not account for local microclimatic variation or anthropogenic habitat modifications—factors that may create artificially suitable environments for invasive species (Xue et al. 2025). Future research should incorporate higher‐resolution environmental variables and consider the role of irrigated agriculture in creating microclimatic refugia, as has been suggested for other invasive pests (Hill et al. 2017).

Our results carry several important implications for *H. halys* management. First, the absence of IBD and IBE signals suggests that phytosanitary measures should prioritize high‐risk invasion pathways—particularly international trade routes and transportation networks—over region‐specific climate‐based alerts. Second, the identification of genetic admixture zones in Greece and Belgium signals that these areas could become sources of particularly adaptable populations, warranting intensive monitoring and rapid response. Third, while insecticide resistance has not yet been documented for *H. halys* (Kuhar and Kamminga 2017), the presence of outlier SNPs near resistance‐associated genes in invasive populations (Parvizi et al. 2023), combined with the standing genetic variation we have documented in admixture zones, suggests that resistance phenotypes may have the potential to evolve. Proactive resistance management strategies, including rotation of insecticide modes of action and integration of behavioral control methods (e.g., pheromone‐based attract‐and‐kill), should be implemented preemptively.

## 5 Conclusion

In conclusion, this study provides a quantitative test of IBD and IBE for the global invasion of *H. halys* , demonstrating that natural dispersal barriers do not explain the observed genetic structure. The absence of both IBD and IBE signals indicates that anthropogenic forces have fundamentally decoupled population genetic structure from natural biogeographic templates. These findings complement recent genomic reconstructions that have detailed the timing and routes of invasion (Boscolo Agostini et al. 2025), converging on a unified conclusion: human‐mediated dispersal—not natural dispersal barriers—is the primary driver of *H. halys* 's global expansion. By quantitatively testing the predictions of natural dispersal models, this study advances our fundamental understanding of invasion mechanisms and underscores the urgent need for globally‐coordinated biosecurity strategies that target human‐mediated dispersal pathways. As international trade continues to expand, the challenge lies in matching the dynamism and scale of anthropogenic dispersal with equally agile, evidence‐based management approaches.

## Author Contributions

**Xiaoyu Yan:** data curation (equal), formal analysis (lead), investigation (equal), methodology (lead), software (equal), visualization (lead), writing – original draft (lead), writing – review and editing (equal). **Yu Chen:** data curation (equal). **Dianyu Liu:** data curation (equal). **Zhihan Su:** data curation (equal). **Wenyan Xu:** data curation (equal). **Yichen Wang:** data curation (equal). **Chenxi Liu:** conceptualization (lead), data curation (equal), funding acquisition (lead), project administration (lead), resources (lead), supervision (lead), writing – review and editing (equal).

## Funding

This study was supported by the Sino‐American Biocontrol International Cooperation Program [59‐0212‐9‐001‐F]. The sponsor played no role in any aspect of this study. The funders had no role in the design of the study; in the collection, analysis, or interpretation of data; in the writing of the manuscript; or in the decision to publish the results.

## Disclosure

No use of AI was reported.

## Ethics Statement

The authors have nothing to report.

## Conflicts of Interest

The authors declare no conflicts of interest.

## Supporting information


**Table S1:** COI sequences of *Halyomorpha halys* sourced from previously published studies.
**Table S2:** COI sequence information and accession numbers of *H. halys* newly downloaded from NCBI and BOLD.
**Table S3:** Sampling sites, sample sizes, and geographic coordinates (latitude and longitude) across 15 countries.
**Table S4:** Climatic data for all sampling regions.

## Data Availability

Data that support the findings of this study are available in the main text or Supporting Information.
